# Exploiting the Fractionation of Stable Isotopes in Biochemical Processes for Medical Diagnosis: A Narrative Review

**DOI:** 10.14336/AD.2024.1577

**Published:** 2026-01-20

**Authors:** Markus A. Hobert, Niklas Helle, Christopher Siebert, Anton Eisenhauer, Martha Gledhill, Walter Maetzler

**Affiliations:** ^1^Department of Neurology, and University Medical Center Schleswig-Holstein, Campus Kiel, Christian-Albrechts-University of Kiel, Kiel, Germany.; ^2^Department of Neurology, University Medical Center Schleswig-Holstein, Campus Lübeck, University of Lübeck, Lübeck, Germany.; ^3^GEOMAR Helmholtz Centre for Ocean Research Kiel, Kiel, Germany.

**Keywords:** stable isotopes, isotope fractionation, medical diagnostics

## Abstract

Analysis of isotope distributions plays a crucial role in medical diagnostics. While radioactive and radiogenic isotopes - those that undergo or result from radioactive decay - are widely used, stable isotopes are less commonly applied despite their significant diagnostic potential. For example, calcium isotope ratio analysis is already commercially utilized for calcium loss and the early diagnosis of osteoporosis. Additionally, analyses of iron, copper, and zinc isotope ratios have been explored in various conditions, including hemochromatosis, Wilson's disease, cancer, Alzheimer's disease, and amyotrophic lateral sclerosis. Altered isotope ratios in these diseases are thought to reflect pathophysiologically relevant processes, making them promising biomarkers. This review provides a comprehensive overview of the current and potential applications of stable isotope analysis in medicine.

## Introduction

Isotopes are different forms of a chemical element with a difference in atomic mass due to a different number of neutrons but always the same number of protons in the atomic nucleus. As a result, their chemical properties are nearly identical. Isotopes are classified as either unstable (also radioactive and radiogenic) or stable. Radioactive isotopes are characterized by a radioactive decay that transforms them into another, potentially stable, isotope of another element. Stable isotopes are not radioactive and therefore do not decay further [1]. The focus of this review is only on the stable isotopes and their application in medical diagnosis.

The distribution and fractionation (i.e. change in relative abundance of different isotopes) of isotopes in biological systems are widely used in biological and earth sciences to analyze chemical, biochemical, and biological processes [2], making them potentially interesting for medical purposes [3, 4]. In medicine, stable isotope ratios have the potential to provide valuable diagnostic information, but to our knowledge, their potential has only been commercially exploited in one case to date [5].

The basic principle is that in the healthy state the isotope ratio(s) in different compartments of the body such as organs and body fluids are relatively stable due to the underlying physicochemical processes that control their distribution. This state is called homeostasis. However, certain diseases, such as osteoporosis [5], can lead to enrichment of lighter or heavier isotopes of the same element in affected body compartment(s). In this example lighter calcium (Ca) isotopes are enriched in the body fluids with respect to healthy individuals [2, 5]. As body fluids connect different organs and there is always an exchange between them, it is possible to draw direct or indirect conclusions about the distribution and fractionation of isotopes in the body by analyzing the isotopic ratios in these fluids. If the isotopic composition of a diseased/affected organ changes, isotopic mass balance requires that the isotopic composition of the adjacent body fluid is affected. If this effect of the changes in the organ on the associated body fluid is large enough, the ratio in the latter will change significantly and can be detected. In addition, in some cases, the loss of an element can lead to a shift in the isotope ratio(s) if molecules with a particular isotope ratio have been removed from a reservoir to the body fluid [5]. There are also disease-specific proteins that have an unusual binding affinity to a certain isotope [6]. Finally, the isotope ratio is also influenced by the diet [7-10].

While radioactive isotopes are widely used in the clinical routine, the analysis of stable isotope ratios is very exotic in medical diagnostics. However, their diagnostic potential is evident, so this narrative review will highlight the medical fields in which they are already implemented, the biochemical mechanisms that lead to isotopic fractionation and the analytical techniques used to quantify the isotope ratios.

**Figure 1.**
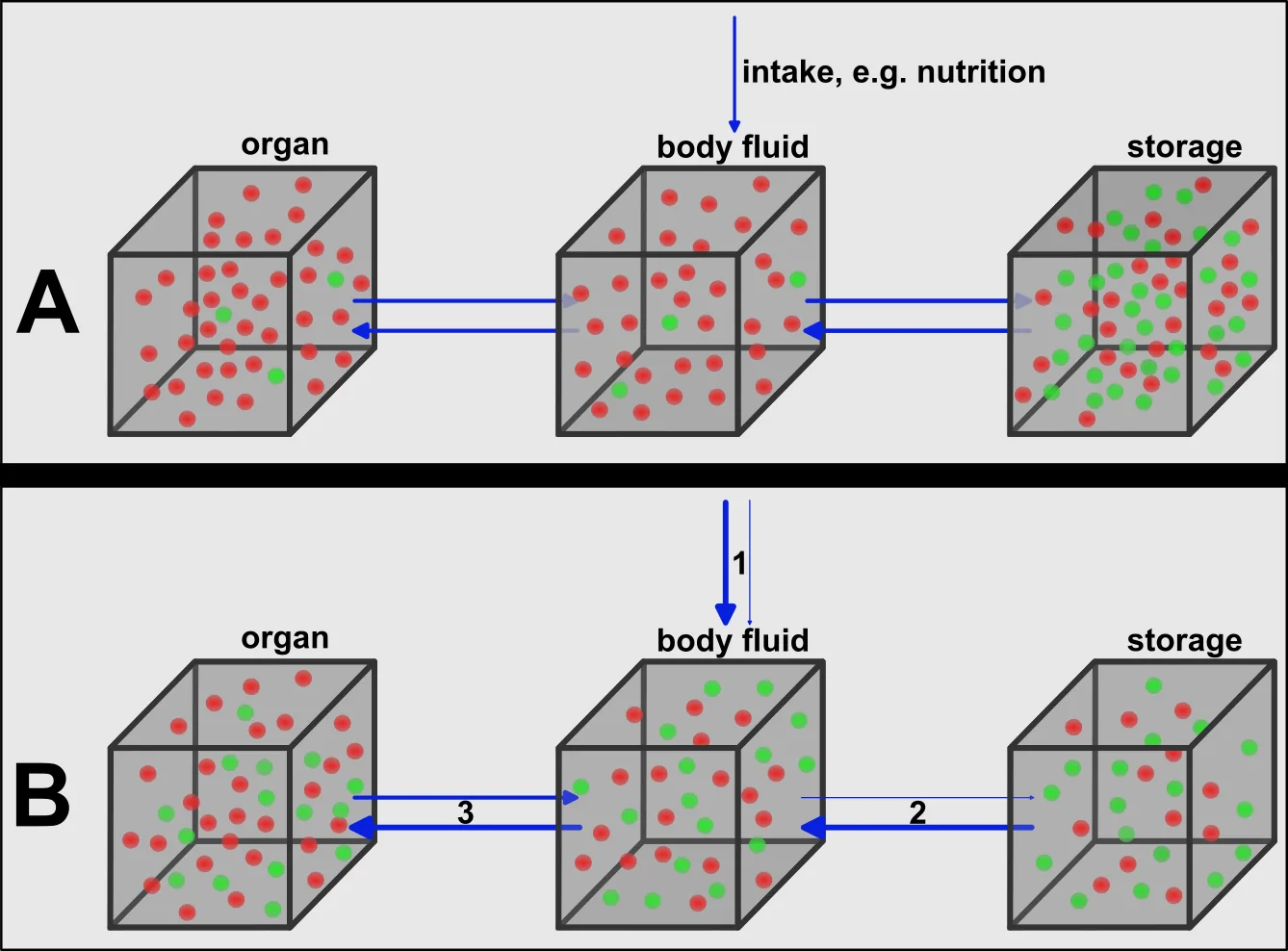
**Overview of isotopic fractionation processes**. Red and green are isotopes of the same element. The three boxes represent different compartments: “organ(s)”, “body fluid(s)” and “storage”. The arrows represent transport processes. (**A**) A steady state is shown. The ratio of green to red is different in each compartment. Here, the green / red ratio is higher in the “organ” compartment than in the “body fluid” compartment. It is highest in “storage”, i.e. fractionation leads to accumulation of the green isotope in the “storage” compartment. (**B**) Mechanisms leading to an altered isotope ratio in the “body fluid” compartment. 1, An increased (in this example of the green isotope) or decreased (in this example of the red isotope) uptake of a particular isotope can alter the green/red ratio in the “body fluid” compartment. 2, when molecules are transferred from “storage” to “body fluid”, the ratio may change due to a different isotopic ratio in the “storage” compartment or due to fractionation during transport processes out of the “storage” compartment. 3, Fractionation due to preferential transport or binding in the “organ” compartment with removal of this isotope from the “body fluid” compartment (red).

## Isotopic fractionation

Isotope fractionation is the process by which isotopes of an element are unequally partitioned or distributed between compartments due to differences in their mass or nuclear properties, often resulting from physical, chemical or biological processes. This results in variations in isotopic ratios that can provide insight into metabolic processes. Isotope fractionation requires at least two compartments for the separation of isotopes in different ratios. In addition, isotopic fractionation requires an incomplete chemical reaction to become measurable. In medical terms compartments are body fluids such as blood, cerebrospinal fluid (CSF), organs, and storage compartments (e.g. liver, bones). In particular, phase transitions from fluid to solid cause fractionation, such as the Ca isotope fractionation during bone formation [11]. From a diagnostic perspective, body fluids such as serum, urine or saliva are of particular interest, because they are relatively easy to access.

Isotopic ratios may differ between the compartments, but as long as the chemical equilibrium or in medical terms the homeostasis is maintained, there is a constant exchange, and the ratios will not change significantly. Depending on the element and body compartment, isotopic ratios can vary because the distribution is determined by the physicochemical distribution mechanisms such as diffusion, transport and or phase transitions. Factors such as dietary habits [7-10], geographical factors [7], age [12], and disease (see references below) can affect the distribution mechanism and thereby the isotope ratio. A schematic overview of important mechanisms from a medical point of view is given in Figure 1. The molecular mechanisms are the preferential binding of an isotope to a protein as a cofactor, in transport processes and in a chemical reaction. An overview is given in Table 1. Diseases that lead to an enrichment [13-16] or depletion of an element [5] drive isotopic fractionation. Protein misfolding diseases, such as neurodegenerative diseases, are also associated with an altered isotopic distribution [6].

**Table 1:** Physicochemical mechanisms leading to isotopic fractionation.

Effect	Description
**Kinetic isotope effect**	Describes a reduced reaction rate of molecules with heavier isotopes, because they move slower and therefore require a higher activation energy. This leads to a preferential reaction of molecules with lighter isotopes and an accumulation of products containing these isotopes [17]. This can lead to an accumulation of the lighter isotope in a chain of chemical reactions or in reactions deriving their isotopes from the depleted (lighter) pool [7].
**Equilibrium isotope effect**	At thermodynamic equilibrium of a chemical reaction, the concentration of each isotope in the products may be different. The magnitude depends on the stability of the molecular species involved and their reactivity, for example a solid product will usually be isotopically heavier than the liquid educt [17].
**Redox reaction**	Describes an exchange of isotopes of the same element during a redox reaction. The mechanism can be an equilibrium effect, a kinetic effect, or a combination of both effects [18].
**Transport processes**	Describes a preference of an isotope in a cellular transport system, e.g. lighter isotopes are often preferred in transport through channels and tight junctions due to the kinetic isotope effect [19].

## Analysis of isotope ratios used for medical diagnostics

Currently, stable isotope ratio analysis is rarely used in routine medical diagnostics. However, their diagnostic power has already been demonstrated in some cases. The studies reported here analyze the isotopic distribution of Ca, iron (Fe), copper (Cu), and zinc (Zn), which are all abundant in the human body [2]. Ca is a major component of bone and Fe, Cu as well as Zn are found as cofactors in several enzymes and transport proteins. Since the storage and distribution of Ca, Fe and Cu are highly regulated, diseases associated with changes in the elemental reservoirs/storages are a logical target for isotopic distribution analysis. Furthermore, from an analytical point of view, these elements are relatively easy to extract and analyze [20, 21].

## Isotope ratios in osteoporosis

Osteoporosis is a disease that leads to an altered metabolism of the bones. It causes bone loss that leads to an increased risk of fractures. The most prevalent form is aging-related, and women are more affected than men. The medical gold standard for diagnosis is by imaging using dual-energy X-ray absorptiometry (DXA) [22]. Recently, however, it has been shown that the composition of Ca isotopes in blood serum and urine, i.e. the ratio of ^44^Ca over ^42^Ca, allows early diagnosis (up to 10 years before DXA) of osteoporosis [5, 23]. This allows a much wider and more effective range of therapeutic measures. In a study of blood and urine from 14 women with osteoporosis and 66 without, a shift in the ^44^Ca/^42^Ca ratio towards the lighter isotope ^42^Ca was found in the diseased group. This is based on the kinetic isotope effect during bone grafting where ^42^Ca is favored and thus the bones are enriched in ^42^Ca over ^44^Ca. When bone loss occurs, as e.g. in osteoporosis, the deposited Ca is dissolved in the blood which causes the above-mentioned shift in the ^44^Ca/^42^Ca ratio [5]. This test is commercially available in Germany. The test procedure can serve as a blueprint for further diagnostic tests using other stable isotopes. The procedure is as follows: a serum and/or urine sample is collected and sent by post to the analytical company. The sample is analyzed by mass spectrometry and a report of the ^44^Ca/^42^Ca ratio is provided to the patient and physician. This allows osteoporosis to be diagnosed at an early stage and very conveniently, just by taking a blood sample (www.osteolabs.de/; accessed 7 Nov 2024).

## Isotope ratios in hemochromatosis

Hemochromatosis is an iron storage disorder. It is caused by a mutation in the HFE gene that leads to an increased absorption of iron in the intestine. The excess iron accumulates mainly in the liver, but also in other organs. If left untreated, it can result in cirrhosis of the liver and other symptoms depending on where the accumulation occurs. Treatment incorporates the reduction of the iron overload by phlebotomy and medication [24].

It has been found that hemochromatosis is associated with a shift in the iron isotope ratio. In a study of blood from 30 patients with hemochromatosis, the ^56^Fe/^54^Fe ratio was higher than in healthy age-matched controls. The magnitude of the ratio correlated with iron accumulation and the severity of hemochromatosis symptoms and was reduced by treatment [15]. The authors explain this finding as follows: The ^56^Fe/^54^Fe ratio is higher in food than in blood. Upon food uptake, the lighter isotope ^54^Fe is favored over ^56^Fe for resorption due to a kinetic isotopic effect with transport proteins. If more iron is resorbed, the pool of light ^54^Fe isotopes becomes depleted and proportionally heavier ^56^Fe isotopes are resorbed instead, resulting in a higher ^56^Fe/^54^Fe ratio in blood. Since in hemochromatosis the iron resorption is malfunctioning, the excessive iron uptake causes an accumulation of the heavier isotope ^56^Fe compared to the healthy population [15].

## Isotope ratios in cancer

Different isotope ratios have been analyzed in different types of cancer. The samples analyzed were mainly tumor tissue, blood and urine. These body fluids are of particular interest because they are easy to collect and have a huge diagnostic potential in the field of early detection, progression and mortality estimation. A selection of examples is described below.

Hepatocellular carcinoma is one of the most common malignant cancers and originates from liver cells. A study investigated the isotopic ratios of copper (^65^Cu, ^63^Cu) and sulfur (^34^S, ^32^S) in blood samples from 23 patients with hepatocellular carcinoma and 20 healthy controls. Patients had higher levels of total copper in serum and in red blood cells than the healthy controls. Moreover, the red blood cells were enriched with the lighter isotope ^63^Cu, while the serum was unaffected. For sulfur, a shift of the isotope ratio towards the lighter sulfur isotope ^32^S was found in red blood cells and also in serum of affected patients [25]. In addition, an analysis of tumor and non-tumor liver tissue from biopsies of seven other patients showed an accumulation of ^65^Cu in the tumor tissue compared to non-tumor tissue [25]. The authors hypothesized that patients with hepatocellular carcinoma have a decreased biliary copper excretion, increased release from copper stores, and an accumulation of ^65^Cu in the tumor. The increased rate of ^32^S in the blood of patients with hepatocellular carcinoma is thought to originate in part from tumor-derived sulfides [25].

The ^65^Cu/^63^Cu ratio has also been investigated in other cancer entities. One study analyzed this isotope ratio in serum samples from 20 breast cancer patients and eight colorectal cancer patients and compared them with data from 50 controls [26]. The authors found lower ^65^Cu/^63^Cu ratios in both breast and colorectal cancer compared to controls. In patients who died during the longitudinal study, the ratio became lower and fell below a certain threshold 3-6 months before currently established tumor markers increased [26].

One study analyzed the ^65^Cu/^63^Cu ratio in the blood of 44 patients with ovarian cancer and 48 healthy controls, as well as tissue from ovarian biopsies with 11 samples of tumor tissue and 10 samples of non-tumor tissue [27]. The authors found a decreased ^65^Cu/^63^Cu ratio in the serum of patients with ovarian tumors compared to controls. In the tumor samples, ^65^Cu was enriched compared to non-tumor tissue.

The isotopic fractionation of Cu in cancer patients leads to an accumulation of the heavier ^65^Cu in the tumor cells and a consequent shift in the ^65^Cu/^63^Cu ratio in the surrounding tissues and body fluids towards the lighter ^63^Cu isotope. The underlying biochemical processes are hypothesized to lead to an increased concentration of lactate in the hypoxic tumor environment. The carboxyl groups of lactates have a high binding affinity for Cu. This binding is more stable to the heavier ^65^Cu than to the lighter ^63^Cu, leading to fractionation when copper is removed from cells into the blood [26, 27].

In another study, the isotopic ratios of Fe, Cu and Zn in plasma were analyzed in a cohort of 47 patients with hematological malignancies and compared with samples from 50 healthy controls [28]. The authors found a lower ratio for ^65^Cu/^63^Cu and a higher ratio for ^66^Zn/^64^Zn in patients compared to controls. A reduced ^65^Cu/^63^Cu and an increased ^66^Zn/^64^Zn ratio were associated with increased mortality. Furthermore, the combination of these markers identified a cohort with poor survival, that was not found by other known laboratory tests. The authors conclude that changes in isotope ratios are useful markers for predicting mortality in patients with hematological tumors [28].

Several studies investigated the use of the ^66^Zn/^64^Zn ratio as marker in cancer [28-31]. One study compared breast cancer tissue, tissue from benign breast tumors (n=16), healthy tissue adjacent to malignant (n=15) and benign tumors (n=7), and healthy breast tissue (n=8) from people without breast cancer [31]. They found a lower ^66^Zn/^64^Zn ratio in tissue from malignant tumors compared to healthy tissue. The ^66^Zn/^64^Zn ratio was also lower in tissue from malignant and benign tumors than in tissue adjacent to benign tumors, but not adjacent to malignant tumors [31].

The isotopic ratio of ^66^Zn/^64^Zn was also investigated in the urine of patients with pancreatic cancer, a malignant tumor that is usually diagnosed at a late stage, as this tumor causes only few symptoms in early stages. Therefore, pancreatic cancer has a high mortality rate. In the urine of 21 patients with this tumor, a higher amount of the lighter isotope ^64^Zn was found compared to the urine of 46 healthy controls [30]. This finding may be useful in detecting pancreatic cancer at an earlier stage.

The same authors extended this approach to other cancers and examined the ^66^Zn/^64^Zn ratio in the urine of in 22 patients with prostate cancer, 16 with breast cancer and 14 with benign breast tumors and compared it with data from 45 healthy controls [29]. The prostate cancer group was further categorized into high, intermediate and low risk of recurrence. They found no significant difference in the ^66^Zn/^64^Zn ratio between breast cancer and healthy controls. The proportion of the lighter ^64^Zn in the urine of patients with benign breast tumors was increased compared to healthy controls. When the urinary ^66^Zn/^64^Zn ratio was compared between patients with prostate cancer and controls, it was lower in patients with prostate cancer, indicating a higher excretion of the lighter ^64^Zn. There was also an association of the risk of recurrence with higher levels of ^64^Zn in patients at high-risk tumors. This parameter may therefore be useful to estimate prognosis and possibly to detect recurrence after treatment [29].

The pathophysiology of Zn in cancer is not fully understood. Considerations include an up-regulation of metallothionein, a protein with a large capacity to bind Zn ions to cysteine. A kinetic isotope effect favors the lighter ^64^Zn, leading to isotopic fractionation [32]. Other mechanisms include up-regulation of Zn transporter proteins [32], increased reactive oxygen species and oxidative stress. These cause an increased release of free Zn from the cysteine ligand, which is thought to be preferentially the lighter ^64^Zn isotope [29, 30].

The advantage of isotope analysis in the field of cancer would be in the early detection of certain tumors (e.g. pancreatic cancer) to start treatment at an earlier stage of the disease. Other applications would be in the estimation of mortality and progression and in the post-treatment monitoring phase for early detection of relapse.

## Isotope ratios in Wilson’s disease

Wilson's disease (WD) is a copper storage disorder. It is caused by mutations in the ATP7B gene, which affects copper metabolism. It leads to an accumulation of copper in the liver, brain, and other organs. If left untreated, liver failure and neurological symptoms can be the outcome. State-of-the-art diagnosis includes determination of ceruloplasmin and copper levels in serum and 24-hour urine, as well as liver biopsy and genetic testing. Treatment consists of chelation therapy of copper to allow urinary excretion [33].

By measuring serum Cu concentration and the ^65^Cu/^63^Cu ratio, one study was able to clearly differentiate between patients with WD (N=5), healthy controls (N=18) and infants (N=2) [34]. In a methodological study, urine samples from three untreated and three treated patients with Wilson's disease and one control were analyzed. The proportion of ^65^Cu was lower in samples from untreated patients compared to treated patients and the control, suggesting a reduced excretion of this isotope [14].

In another study, the ^65^Cu/^63^Cu ratio in the whole blood of 36 patients with WD was compared with that of 75 healthy control subjects. No difference was found between untreated patients and controls. However, the data for a treatment effect were inconsistent: While a shift of the Cu isotope ratio towards the lighter one (^63^Cu) was found in treated patients compared to untreated ones and controls, a longitudinal analysis of a sub-sample of 8 (treated) patients showed no treatment effect on the isotope ratio [13].

In addition, a lower ^65^Cu/^63^Cu ratio and hence a relative accumulation of ^63^Cu was associated with more advanced hepatic symptoms. Much lower values were found in patients with neurological symptoms. The authors suggested that the ^65^Cu/^63^Cu ratio has potential as a diagnostic and prognostic marker for the degree of liver injury [13].

The reason for the fractionation of Cu isotopes in WD is unknown. A mouse model in which the ^65^Cu/^63^Cu ratio in serum, liver and feces was studied longitudinally showed a decreasing ratio over time, indicating an accumulation of ^63^Cu. A possible mechanism is an increased uptake of ^63^Cu into cells via a transporter protein. This increased uptake is driven by a preferential reaction of an enzyme with the lighter ^63^Cu in a redox reaction prior to transport. This enzyme also suppresses the excretion of bound ^63^Cu [13].

Therefore, there may be some potential here to use isotope analysis for early detection and treatment monitoring of this disease, but further studies are needed due to inconsistencies in the available studies.

## Isotope ratios in Alzheimer’s disease

Alzheimer's disease (AD) is the most prevalent neurodegenerative disease, leading to cognitive decline and eventually to dementia. Although the patho-physiology is not fully understood, deposits of tau and amyloid beta (Abeta) protein are hallmarks of AD [35].

Post-mortem studies of the frontal cortex of 10 patients with Alzheimer’s disease and 10 controls revealed an enrichment of ^63^Cu for AD patients. For zinc (Zn) the ^66^Zn/^64^Zn ratio was higher in AD patients indicating increased ^66^Zn levels for them. Both isotope shifts become more pronounced as the disease progresses, as a correlation with Braak stages [36] has been found.

In a mouse model of AD (APPswe/PSEN1dE9 transgenic mice), a non-significant trend for the enrichment of the isotopically lighter ^63^Cu was found in brain tissue of AD mice compared to the wild-type (WT) mice. In contrast, the serum of AD mice was enriched in ^65^Cu compared to the WT mice. The authors attribute these observations to an as yet unknown mechanism of isotope fractionation during the proliferation of amyloid-beta plaques in the brain [37]. In the same mouse model an enrichment of ^66^Zn was found in the brains of AD mice compared to WT [38], confirming the findings from the human brains [6]. This difference was found at a time point after the onset of Abeta deposition. No differences were found in serum and erythrocytes. The authors account the isotope fractionation for zinc in the AD brains to an inverse kinetic isotope effect (preferential binding of the heavier ^66^Zn isotope) during Abeta plaque proliferation, leading to an enrichment of ^66^Zn in the plaques [38].

Another study found a depletion of ^66^Zn in the CSF of AD patients (N=14) compared to healthy controls (N=11) [39]. Since CSF is the adjacent fluid to the brain, the authors account their observations to the above-mentioned inverse kinetic isotope effect and the enrichment of ^66^Zn in brain tissue [6, 38] at the expense of the amount of ^66^Zn in CSF. With regard to copper, no significant differences were found between the CSF of AD patients and controls [39].

The biochemical mechanisms of isotopic fractionation in AD are most likely caused by aggregation of Abeta and tau, since both proteins interact with metals, i.e. Zn and Cu ions. In healthy brains, Cu is bound to cysteine as Cu^2+^, but to Abeta as Cu^+^. The redox reaction that reduces Cu^2+^ to Cu^+^ favors the isotopically lighter ^63^Cu, leading to isotopic fractionation. In addition, Cu^+^ binds to histidine on tau proteins, also contributing to isotopic fractionation [6, 38].

Zn also binds to cysteine in healthy brains. In the brains of AD patients, Zn accumulates due to the preferential binding of Zn^2+^ to three histidine residues of Abeta proteins. Isotopic fractionation occurs due to a kinetic isotope effect. This is also enhanced by the stronger binding of Zn to histidine than to cysteine [6, 38].

## Isotope ratios in amyotrophic lateral sclerosis

Amyotrophic lateral sclerosis (ALS) is a rare neurodegenerative disease that affects the first and second motor neurons, leading to progressive muscle weakness. There is currently no cure for ALS in most cases [40].

One study investigated the isotope ratios ^65^Cu/^63^Cu and ^66^Zn/^64^Zn in the cerebrospinal fluid of 31 patients with ALS, 11 healthy controls and 14 patients with Alzheimer's disease. The ^65^Cu/^63^Cu ratio was higher in patients with ALS compared to healthy controls, i.e. the proportion of the heavier isotope ^65^Cu was higher. The comparison of the same parameters between ALS and AD was not significant. Regarding the distribution of Zn isotopes, no significant difference was found between ALS and the other two groups. The discriminative ability of the ^65^Cu/^63^Cu ratio to distinguish ALS from controls was 76% in a ROC analysis [39]. The biochemical mechanisms of isotopic fractionation in ALS are unknown. The authors hypothesized that the lower levels of ^63^Cu are best explained by a preferential binding of this isotope to aggregated proteins in the brain via a kinetic isotope effect. Since brain and CSF are interacting compartments, an accumulation of ^63^Cu in the brain tissue leads to a decrease in CSF. Another suggestion from the authors is based on the metal loading of superoxide dismutase 1 (SOD1), an enzyme that is pathophysiologically linked to ALS [40]. In a healthy state, the enzyme contains two copper and two zinc ions but in ALS, SOD1 loses the copper ions. In a mouse model, it was shown that only copper-depleted SOD1 is able to form aggregates, whereas copper-rich SOD1 remains soluble [41]. When aggregation of immature SOD1 occurs, as in ALS, an over-metallization of the remaining soluble SOD1 may occur. This over-metallization could lead to isotopic fractionation [39].

In contrast, another study using an ALS mouse model, brain, spinal cord and muscle tissue, as well as blood and food/feces from mice with a G93A mutation of human SOD1 were compared to wild-type mice. Mice were sacrificed at specific time points to track changes over a period of up to 120 days. They found no significant differences in the ^65^Cu/^63^Cu ratio between the two groups at any of the timepoints studied [42].

## Experimental techniques for stable Isotope ratios

Due to very similar physicochemical properties of the isotopes of an element, isotope ratios can only be determined by means mass spectrometry. Mass spectrometry uses the mass to charge (m/z) ratio of analytes (in this case isotopes of an element) in the gas phase in a vacuum to separate, detect, and quantify them. The ionization source serves to create a stream of charged ions that can be transferred into the vacuum compartment of the mass spectrometer. Apart from the ionization source, a mass spectrometer also contains a mass analyzer where the ions are separated and an ion-sensitive detector. Out of the multitude of commercially available mass spectrometers, only a multi-collector inductively-coupled plasma mass spectrometer (MC-ICP-MS) is suitable for analysis of isotopes heavier than mass 36 with sufficient accuracy, precision and sample throughput capacity. The principal function of a MC-ICP-MS is described below.

To obtain precise and accurate results the element sample pretreatment is required for the removal of organic material. In addition, the element in question must be chemically separated from the rest of the inorganic sample components (the sample matrix).

The organic material in a sample is usually removed through a “digestion process”. Organic material is broken down at elevated temperatures (ca. 100-120°C) using strong oxidizing mineral acids such as nitric acid. The resultant oxidized carbon is then removed by evaporation. This process is commonly performed in non-reactive Teflon vials, either on a hotplate or, more efficiently, in a specially designed microwave digestion oven [43].

After removal of the organic material, the element in question is separated from the inorganic sample matrix via ion exchange chromatography. In this process a liquid with the sample in solution is passed through a column filled with an ion exchange resin. A mixture of (or a single) ion species is absorbed to the resin and can subsequently be eluted selectively. Such resins consist of, for example, styrene beads with attached functional organic groups. These groups are highly selective in their adsorption behavior, so that the type of resin (e.g. anion or cation exchange resins) can be altered to target particular analytes of interest [44]. Each element therefore requires a bespoke approach for separation from the sample matrix, and such approaches can range from short and simple (hours) to long and complex (several days). For these reasons, this procedure is still often performed manually, limiting the possible sample throughput. Since significant changes in isotope ratios are still very small, even the smallest contaminations in the preparation process must be prohibited and a yield of more than 95 % is required to prevent isotopic fractionation which makes well-trained personnel indispensable for reliable and reproducible results. However, automated systems are becoming increasingly more available [45] which will make stable isotope ratio analysis accessible for a much broader community.

Ideally, after ion exchange chromatography only the element in question will be left in solution and can be transferred into the mass spectrometer.

As described above, transfer through the mass spectrometer, mass separation and detection require the element to be analyzed to be a gaseous ionic form. Therefore, the sample solution is first introduced into the ionization source, the inductively-coupled plasma (ICP).

**Figure 2.**
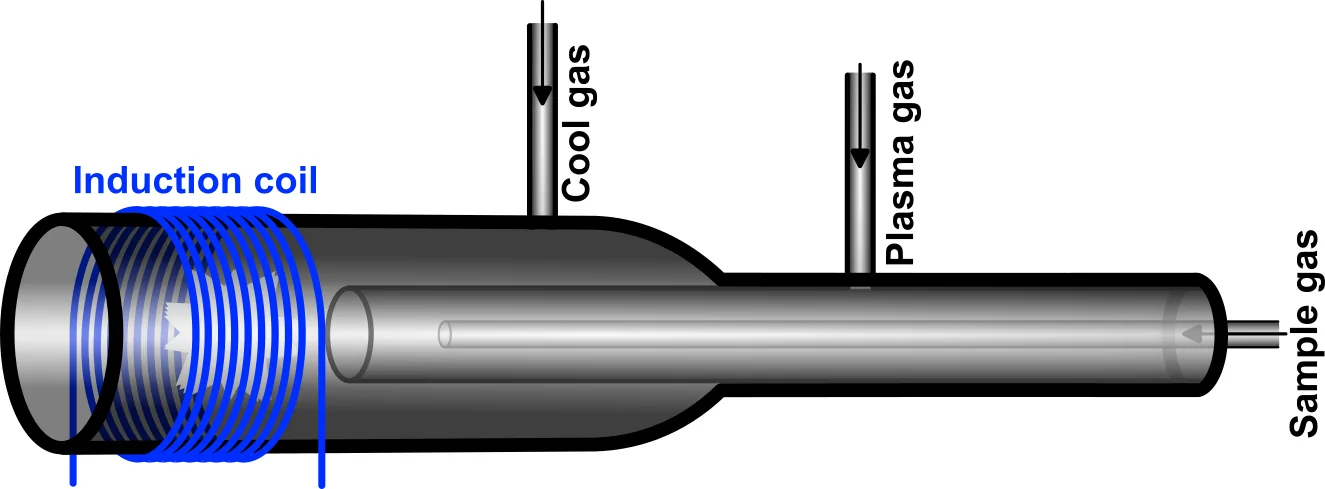
**Schematic depiction of an ICP torch**. The plasma gas (Ar) is ignited by a spark which is then inductively coupled with the RF-modulated magnetic field from the induction coil to generate the plasma. The analyte is transferred by the sample gas into the plasma where the compounds are first atomized and then ionized. The cool gas protects the fused silica from plasma.

Generally, in physics plasma is, besides solid, liquid and gaseous, the fourth state of matter. It is characterized by very high temperatures of a gas which cause the outer electrons of any ions in the plasma to separate from the atomic nuclei and become ionized [46]. Introduction of an analyte into the plasma will cause the analyte to also become ionized. The analyte ions can then be detected with a mass spectrometer.

For the ICP the noble gas Argon (Ar) is used to generate the plasma due to its high ionization potential (IP = 15.76 eV) [47], non-toxic properties and ready availability. The ICP is generated in a torch (*cf.* Fig. 2) with three cylinders made of fused silica: The inner one for the sample gas that carries the analyte into the plasma, the middle one for the plasma gas and the outer one for the cool gas to keep the plasma away from the outer glass walls.

Upon contact with the ICP, almost any compound will fall apart in its individual atoms which are subsequently ionized (M^+^).

**Figure 3.**
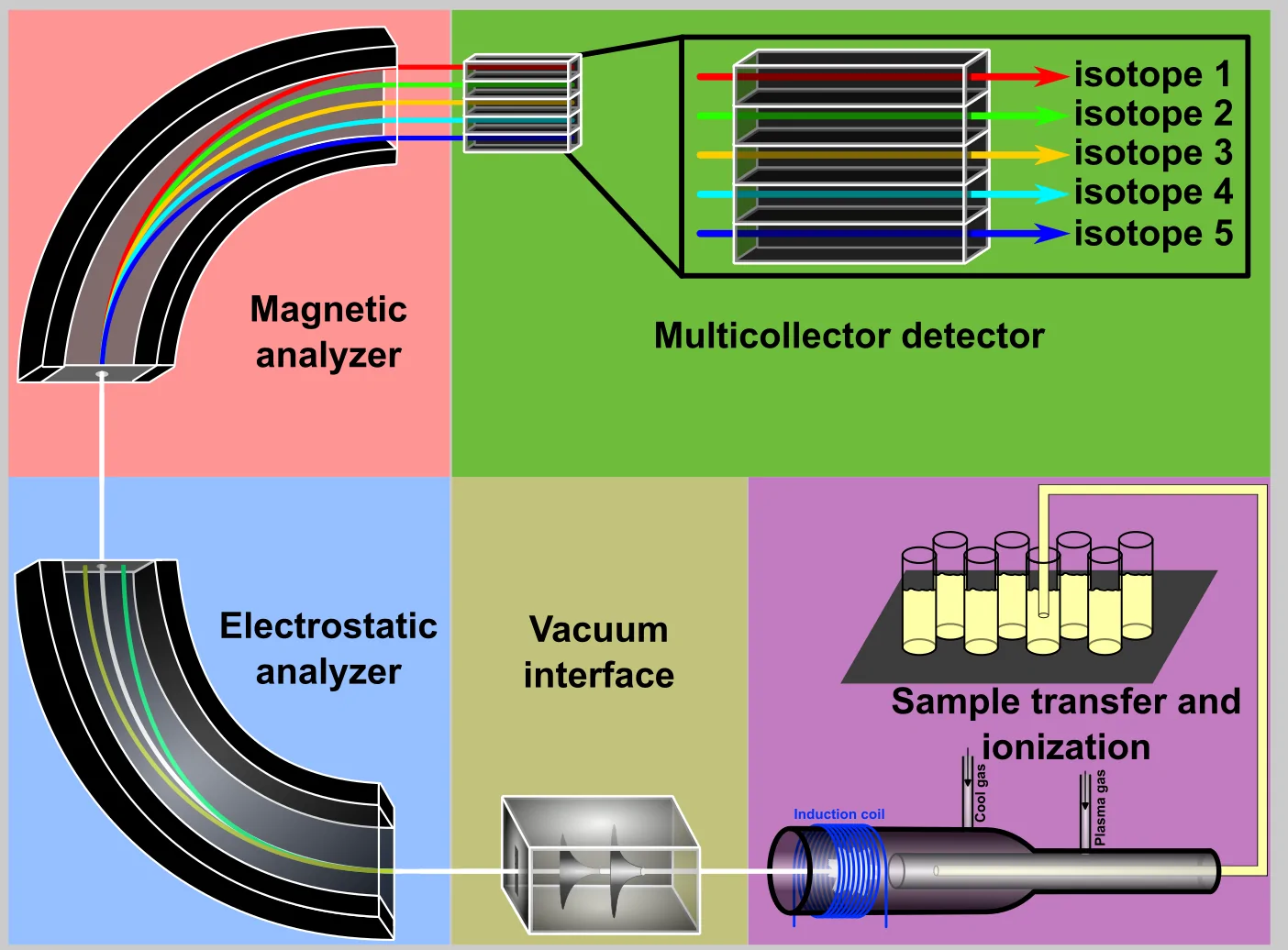
Schematic description of a multicollector inductively coupled plasma mass spectrometer for isotope-ratio mass spectrometry.

Since this occurs at ambient pressure but mass spectrometers usually operate in the (high) vacuum, the ions have to be transferred through an interface. This is shown in the bottom middle of Figure 3. A double-focusing mass analyzer is located after the ionization source. In the first part (bottom left of Fig. 3) the ions enter the electrostatic analyzer (ESA) where they are affected by a static electric field. Since the ESA is bent, the voltage can be set so that only the m/z ratios of interest pass through. In the second part (top left of Fig. 3), this beam of survivor ions enters the magnetic analyzer where the ions interact with a perpendicular magnetic field. The Lorentz force causes the ions of different m/z ratios to exit the magnetic analyzer at different positions. By placing multiple detectors at the end (top right), one is able to quantify the isotopes of interest and also their isotopic ratio at the same time.

Analysis of isotopes by MC-ICP-MS has several advantages that facilitates wide application within the field of geochemistry and could be usefully exploited in applications within medicine: The isotopic ratio of the elements of interest in a given sample compartment is established in vitro. Secondary reactions after sampling (e.g. oxidation of organic matter) will not affect this established ratio once the compartment is isolated. All isotopic ratios are analyzed and calculated relative to an internationally certified, commercially available standard that runs with each batch of samples. This normalization ensures that results from different laboratories, measured under different conditions and at different times, are still comparable in absolute terms. The precision and accuracy of the method is also determined by the repeat analysis of a (certified) standard material. This allows interpretation of isotopic differences between groups (e.g. apparently healthy - diseased) with statistically relatively small sample sets. Another advantage is, that the analysis of isotopic distribution is not dependent on the amount and concentration of an element and may be therefore a reliable biomarker [39].

Despite all these advantages, the requirements for such analyses must also be mentioned: An MC-ICP-MS is a large, freestanding instrument that, depending on the manufacturer, can take up more than 10 m², which cannot be easily integrated into most existing analytical laboratories. In addition, sample preparation and analysis must take place in a well air- and temperature-conditioned clean room to prevent metal contamination. In this respect, not only the preparation (see above) but also the analysis must be carried out by well-trained personnel, as the instrument settings are extremely critical for obtaining reliable data. Apart from these requirements, the initial cost of an MC-ICP-MS is high (> 700 000-1 000 000 €), but the running costs are also considerable, mainly due to the energy and argon consumption (approx. 30 m³/d), resulting in analysis costs per sample of 50-100 €. These limitations will be one reason why stable isotope ratios are not yet widely used in medical diagnostics, but as described above, their potential as robust biomarkers have been recognized.

## Future research directions

Research into the application of stable isotope analysis for medical diagnostics should continue. Further studies are needed in several directions:

On the analytical side, further development of techniques would advance the field, such as making instruments smaller and cheaper for a wide range of applications. There are already promising developments at the prototype level [48, 49]. A deeper understanding of the specific and disease-related mechanisms is still lacking in most cases. This would allow a deeper understanding of disease processes and bring this promising approach closer to clinical application. Therapeutic approaches targeting e.g. metal metabolism are also possible [50]. As some of the results are heterogeneous, further studies should replicate or falsify these experiments. If the results are valid, larger prospective studies are also needed to test diagnostic sensitivity and specificity and to determine cut-off values, paving the way for implementation in clinical routine. As the knowledge of isotope diagnostics in the field of cancer seems to be broad, as promising results as prognostic and early detection markers have been found [26, 28-30], this could be of particular interest for introduction into patient care. As there is a need for biomarkers, research should be extended to other diseases. In particular, the analysis of isotopes in Parkinson's disease is of interest, as Fe seems to play a role in the pathophysiology [51].

## Conclusion

Research in this area focuses primarily on Ca, Fe, Cu, and Zn because these elements play important roles in human metabolism, are relatively abundant in the human body, and are relatively easy to measure. The commercial application of the Ca isotope ratio in the diagnosis of osteoporosis demonstrates the potential of stable isotope analysis in the medical field. The focus on Fe and Cu is probably due to the relatively close mechanisms involved in the storage diseases hemochromatosis and Wilson's disease. In addition, Fe, Cu and Zn serve as essential cofactors in numerous enzymes and may exhibit disease-specific isotopic variations. Determining the isotopic distribution of various elements can provide valuable information about metabolic and disease mechanisms in the human body. Although diagnostic applications of this method are not yet widely used in medicine, changes in isotopic ratios - particularly for Cu and Zn - have been observed in several diseases, including neurodegenerative disorders such as Alzheimer's disease and amyotrophic lateral sclerosis. These isotope shifts are often associated with pathophysiologically relevant processes. Combining isotope distribution analysis with studies of proteins and their modifications holds great potential for unraveling disease mechanisms. This approach can serve as a powerful biomarker for disease diagnosis, monitoring of disease progression, and evaluation of therapeutic response.
